# Detecting and correcting the bias of unmeasured factors using perturbation analysis: a data-mining approach

**DOI:** 10.1186/1471-2288-14-18

**Published:** 2014-02-05

**Authors:** Wen-Chung Lee

**Affiliations:** 1 Research Center for Genes, Environment and Human Health, College of Public Health, National Taiwan University, Rm. 536, No. 17, Xuzhou Rd., Taipei 100, Taiwan; 2 Institute of Epidemiology and Preventive Medicine, College of Public Health, National Taiwan University, Rm. 536, No. 17, Xuzhou Rd., Taipei 100, Taiwan

**Keywords:** Epidemiologic methods, Confounding, Data mining, Effect modification, Bias, Standardization

## Abstract

**Background:**

The randomized controlled study is the gold-standard research method in biomedicine. In contrast, the validity of a (nonrandomized) observational study is often questioned because of unknown/unmeasured factors, which may have confounding and/or effect-modifying potential.

**Methods:**

In this paper, the author proposes a *perturbation test* to detect the bias of unmeasured factors and a *perturbation adjustment* to correct for such bias. The proposed method circumvents the problem of measuring unknowns by collecting the perturbations of unmeasured factors instead. Specifically, a perturbation is a variable that is readily available (or can be measured easily) and is potentially associated, though perhaps only very weakly, with unmeasured factors. The author conducted extensive computer simulations to provide a proof of concept.

**Results:**

Computer simulations show that, as the number of perturbation variables increases from data mining, the power of the perturbation test increased progressively, up to nearly 100%. In addition, after the perturbation adjustment, the bias decreased progressively, down to nearly 0%.

**Conclusions:**

The data-mining perturbation analysis described here is recommended for use in detecting and correcting the bias of unmeasured factors in observational studies.

## Background

**Figure 1:**
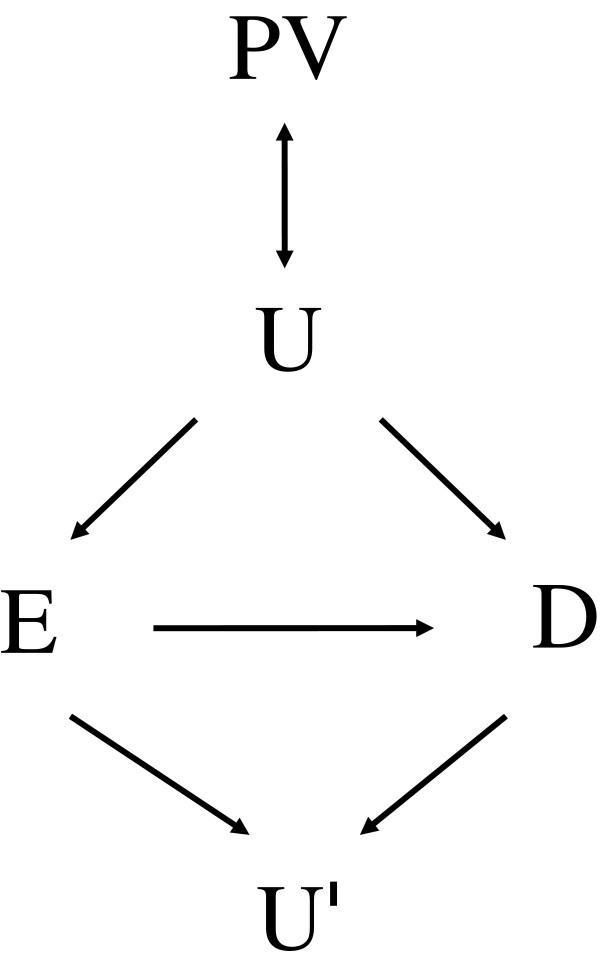
Relations between exposure (E), disease/outcome (D), unmeasured factor with confounding and/or effect modifying potential (U), perturbation variable (PV), and collider (U′).

The randomized controlled study is the gold-standard research method in biomedicine. In contrast, the validity of a (nonrandomized) observational study is often questioned because of factors that are not measured in the study [1]. An unmeasured factor can produce a confounding bias if it is associated with the studied exposure and disease simultaneously. An unmeasured factor can also exhibit effect modification; the exposure-disease relationships are different depending on the presence or absence of the unmeasured factor or on the different levels of intensity. Figure 1 presents the relationships among exposure (E), unmeasured factor (U), and disease (D).

Correcting the bias of a factor with the confounding and effect-modifying potential shown in Figure 1 presents no major challenge. Here, techniques such as standardization should work well [1]. To perform the correction, factors with biasing potential must be identified and measured in the study. However, this is often not possible due to limited knowledge of what these factors might be or, if we have knowledge of them, the cost constraint of actually measuring them.

This paper presents a novel method, termed perturbation analysis, to detect and correct the bias of unmeasured factors. The method circumvents the problem of measuring unknowns by collecting the perturbations of unmeasured factors instead. A perturbation variable (PV) is a variable that is readily available, or can be measured easily, and is potentially associated, though perhaps only very weakly, with U (Figure 1). Note that a PV is associated with E and D only through U (Figure 1). If this is not the case, then the variable by itself is a classical confounder for the E-D relationship and can be adjusted for as such.

As an example, E is asbestos, D is lung cancer, and U is smoking status (unmeasured in the study). Then, PV can be anything not known to be associated with asbestos exposure and lung cancer, but may be associated with smoking status (causally or noncausally, directly or indirectly, positively or negatively), such as personality traits, finger color, breath odor, accessibility to convenience stores, internet usage records, driving records, etc. As another example, E is electromagnetic radiation, D is childhood leukemia, but U is utterly unknown (or perhaps nonexistent). Here, we may try virtually any variable.

However, care must be taken not to include any variable that is associated with the collider of the E-D association. A collider, the U′ in Figure 1, is an effect/consequence of both E and D [1]. Controlling a collider (or its perturbations) can aggravate the bias instead of reducing it. To avoid this, one can collect only those PVs measured before D occurs. If all the PVs in a study precede D, the causal temporality principle dictates that no PV can be associated with the colliders of the E-D association.

The central tenet of the proposed perturbation analysis is to collect a great number of PVs, i.e., hundreds, thousands, or even more. The quickest way to obtain large numbers of admissible PVs is to put in all the questionnaires and laboratory data that has been collected or measured before D occurs. Another possibility is through record linkage of the study subjects to large existing databases, e.g., data pertaining to health insurance, traffic violations, internet usage, etc., where a great number of variables can be found or defined preceding the study outcome [2]. If the subjects in one study are also taking part in genome-wide association studies, the wealth of genomic data (thousands or even millions of genetic markers) could then provide yet another rich source for admissible PVs, particularly because genes can be considered to precede any outcome studied. Essentially, the method represents a data-mining approach.

## Methods

### Bias of unmeasured factors

Before introducing the method of perturbation analysis, we need a metric to quantify the bias of unmeasured factors [3]. There are three variables involved: a binary exposure (E), a binary disease/outcome (D), and a polytomous variable (U), which represents the cross-tabulations of all unmeasured factors. Assume that U has a total of *L* (*L* > 1) levels (indexed by *i*). In the *i*th level, we let *m*_*i*_ denote the number of subjects, *p*_*i*_ denote the exposure prevalence [*p*_*i*_ = Pr(E = 1|U = *i*)], *q*_*i*_ denote the exposure odds [*q*_*i*_ = *p*_*i*_/(1 − *p*_*i*_)], $r_{i}^{u}$ denote the disease risk for an unexposed subject [$r_{i}^{u}=\mathrm{Pr}(D=1\left|E=0,U=i)\right)$], and $r_{i}^{e}$ denote the disease risk for an exposed subject [$r_{i}^{e}=\mathrm{Pr}(D=1\left|E=1,U=i)\right)$]. In the population as a whole, the exposure prevalence is $\bar{p}=\frac{\sum_{i}m_{i}p_{i}}{\sum_{i}m_{i}},$ the exposure odds is $\bar{q}=\frac{\bar{p}}{1-\bar{p}},$ the disease risk in an unexposed subject is ${\bar{r}}^{u}=\frac{\sum_{i}m_{i}\left(1-p_{i}\right)r_{i}^{u}}{\sum_{i}m_{i}\left(1-p_{i}\right)}$, the disease risk in an exposed subject is ${\bar{r}}^{e}=\frac{\sum_{i}m_{i}p_{i}r_{i}^{e}}{\sum_{i}m_{i}p_{i}},$ and the crude risk ratio is

$$\text{crude}\;\text{RR}=\frac{{\bar{r}}^{e}}{{\bar{r}}^{u}}.$$

The standardized risk ratio (SRR) with the total population taken as the standard is the focal point of this paper and can be calculated as follows:

(1) $$\text{SRR}=\frac{\sum_{i=1}^{L}m_{i}r_{i}^{e}}{\sum_{i=1}^{L}m_{i}r_{i}^{u}}.$$

The numerator in [1] represents the total number of subjects who would have contracted the disease if the whole population were exposed, whereas the denominator is the total number of diseased subjects if the whole population were to be unexposed. As such, the index of SRR represents the causal effect of the exposure in the population at large. However, an observational study with U unmeasured does not permit a calculation of the index.

The bias of using the observed crude RR as a substitute for the unknown SRR can be quantified using an equation. Additional file 1: Supplementary Appendix 1 shows that the confounding risk ratio (CRR) is $\text{CRR}=\frac{\text{crude}\;\text{RR}}{\text{SRR}}=\frac{1+\bar{p}\times \sigma_{\text{EOR},\;{\text{DRR}}^{u}}}{1+\left(1-\bar{p}\right)\times \sigma_{{\text{EOR}}^{-1},\;{\text{DRR}}^{e}}},$ where $\sigma_{\text{EOR},\;{\text{DRR}}^{u}}$ is a weighted covariance between the exposure odds ratios (EORs) and the disease risk ratios for the unexposed (DRR^*u*^s), and $\sigma_{{\text{EOR}}^{-1},\;{\text{DRR}}^{e}}$ is a weighted covariance between the inverse of the EORs and the disease risk ratios for the exposed (DRR^*e*^s). Taking a logarithm on both sides of the equation, we arrive at:

(2) $$\begin{array}{l} \log\text{SRR}=\log\text{crude}\;\text{RR}\\ \;+\log\;\left[1+\left(1-\bar{p}\right)\times \sigma_{{\text{EOR}}^{-1},\;{\text{DRR}}^{e}}\right]-\log\;\left[1+\bar{p}\times \sigma_{\text{EOR},\;{\text{DRR}}^{u}}\right]. \end{array}$$

If across the different levels of a U, an increase in exposure prevalence is always associated with an increase in disease risk (see left panel in Table 1), we will have: $\sigma_{\text{EOR},\;{\text{DRR}}^{u}}>0$ and $\sigma_{{\text{EOR}}^{-1},\;{\text{DRR}}^{e}}<0$ (and also $\sigma {'}_{\text{EOR},\;{\text{DRR}}^{u}}>0$ and $\sigma {'}_{{\text{EOR}}^{-1},\;{\text{DRR}}^{e}}<0$ in the next section). From [2], we see that such a U is positively confounding, with crude RR > SRR. On the other hand, if an increase in exposure prevalence is always associated with a decrease in disease risk (right panel in Table 1), we will have a negatively confounding U ($\sigma_{\text{EOR},\;{\text{DRR}}^{u}}<0$ and $\sigma_{{\text{EOR}}^{-1},\;{\text{DRR}}^{e}}>0$, and also $\sigma {'}_{\text{EOR},\;{\text{DRR}}^{u}}<0$ and $\sigma {'}_{{\text{EOR}}^{-1},\;{\text{DRR}}^{e}}>0$), with crude RR < SRR. If there is no variation in the exposure prevalence (middle panel in Table 2) in the disease risk (right panel in Table 2) or in both (left panel in Table 2) across different levels of U, then $\sigma_{\text{EOR},{\text{DRR}}^{u}}=\sigma_{{\text{EOR}}^{-1},\;{\text{DRR}}^{e}}=0$ (and also $\sigma {'}_{\text{EOR},\;{\text{DRR}}^{u}}=\sigma {'}_{{\text{EOR}}^{-1},\;{\text{DRR}}^{e}}=0$). According to [2], there is no bias, and crude RR = SRR.

**Table 1:** A hypothetical population with positive/negative confounding U

**Level of U (*i*)**	**Population number (*m***_***i***_**)**	**Positive confounding**			**Negative confounding**		
		**Exposure prevalence (*p***_***i***_**)**	**Disease risk among the unexposed (**$r_{i}^{u}$**)**	**Relative risk (**$r_{i}^{e}/r_{i}^{u}$**)**	**Exposure prevalence (*p***_***i***_**)**	**Disease risk among the unexposed (**$r_{i}^{u}$**)**	**Relative risk (**$r_{i}^{e}/r_{i}^{u}$**)**
1	2,500	0.76	0.6667	1.2632	0.24	0.4737	1.7593
2	2,500	0.60	0.4000	1.1667	0.40	0.3333	2.4000
3	2,500	0.40	0.3333	1.2000	0.60	0.2000	2.3333
4	2,500	0.24	0.1579	1.0556	0.76	0.1667	1.8947
Total	10,000	Crude relative risk (crude RR) = 1.75			Crude relative risk (crude RR) = 1.53		
		Standardized relative risk (SRR) = 1.20			Standardized relative risk (SRR) = 2.06		

**Table 2:** A hypothetical population without bias

**Level of U (*i*)**	**Population number (*m***_***i***_**)**	**U is associated with neither E nor D**			**U is not associated with E**			**U is not associated with D**		
		**Exposure prevalence (*p***_***i***_**)**	**Disease risk among the unexposed (**$r_{i}^{u}$**)**	**Relative risk (**$r_{i}^{e}/r_{i}^{u}$**)**	**Exposure prevalence (*p***_***i***_**)**	**Disease risk among the unexposed (**$r_{i}^{u}$**)**	**Relative risk (**$r_{i}^{e}/r_{i}^{u}$**)**	**Exposure prevalence (*p***_***i***_**)**	**Disease risk among the unexposed (**$r_{i}^{u}$**)**	**Relative risk (**$r_{i}^{e}/r_{i}^{u}$**)**
1	3,000	0.40	0.30	1.50	0.40	0.40	1.54	0.80	0.30	1.50
2	2,500	0.40	0.30	1.50	0.40	0.30	1.52	0.60	0.30	1.50
3	2,500	0.40	0.30	1.50	0.40	0.25	1.45	0.40	0.30	1.50
4	2,000	0.40	0.30	1.50	0.40	0.20	1.42	0.20	0.30	1.50
Total	10,000	Crude relative risk (crude RR) = 1.50			Crude relative risk (crude RR) = 1.50			Crude relative risk (crude RR) = 1.50		
		Standardized relative risk (SRR) = 1.50			Standardized relative risk (SRR) = 1.50			Standardized relative risk (SRR) = 1.50		

Note that the above analysis of bias (the presence/absence of bias and its direction, if present) is in agreement with what was predicted from the potential-outcome model [4,5].

### Effects of the adjustment of a binary perturbation variable

In the previous section, U is unmeasured and cannot be standardized on in actual practice. It is tempting to adjust for (standardize on) a PV (Figure 1) that is readily available. Assuming that a PV has a total of *V* (*V* > 1) levels (indexed by *j*), the computing formula is:

(3) $$\text{adjusted}\;\text{RR}=\frac{\sum_{j=1}^{V}n_{j}s_{j}^{e}}{\sum_{j=1}^{V}n_{j}s_{j}^{u}},$$

where, at the *j* th level of the PV, *n*_*j*_ is the number of subjects, $s_{j}^{e}$ is the disease risk for an exposed subject [$s_{j}^{e}=\mathrm{Pr}(D=1\left|E=1,\text{PV}=j)\right)$], and $s_{j}^{u}$ is that for an unexposed subject [$s_{j}^{u}=\mathrm{Pr}(D=1\left|E=0,\text{PV}=j)\right)$].

### Theoretical analysis

We now examine the effects of the adjustment of a binary PV theoretically. Let *μ*_PV_ and $\sigma_{\text{PV}}^{2}$ denote the mean and variance of the prevalence of PV across different levels of U, respectively. Using Taylor series expansion, Additional file 1: Supplementary Appendix 2 shows that the expected values of the log adjusted RR (after adjusting for the PV) and the log crude RR are related through the following equation:

(4) $$\begin{array}{l} E\left(\log\text{adjusted}\;\text{RR}\right)\approx \log\text{crude}\;\text{RR}\\ \;+\left(a\times \sigma {'}_{{\text{EOR}}^{-1},\;{\text{DRR}}^{e}}-b\times \sigma {'}_{\text{EOR},\;{\text{DRR}}^{u}}\right)\times f_{\text{PV}}, \end{array}$$

where $\sigma {'}_{{\text{EOR}}^{-1},\;{\text{DRR}}^{e}}$ and $\sigma {'}_{\text{EOR},\;{\text{DRR}}^{u}}$ again are weighted covariances (the primes indicate that they do not adopt the same weights as in the previous $\sigma_{{\text{EOR}}^{-1},{\text{DRR}}^{e}}$ and $\sigma_{\text{EOR},\;{\text{DRR}}^{u}}$, respectively), *f*_PV_ is the ‘variance fraction’ of the PV: $f_{\text{PV}}=\frac{\text{variance in the prevalence of PV across different levels of U}}{\mathrm{total variance}}=\frac{\sigma_{\text{PV}}^{2}}{\mu_{\text{PV}}\times \left(1-\mu_{\text{PV}}\right)}$, and *a* and *b* are two positive constants of less interest.

From [4], we see that adjusting for a PV where *f*_PV_ = 0 (an uninformative PV) is not useful: E(log adjusted RR) = log crude RR. However, adjusting for a PV with *f*_PV_ > 0 (an informative PV) will, on average, push the log adjusted RR away from the log crude RR. Moreover, the direction of this movement correctly indicates where the unknown log SRR might be, i.e., in general we have E(log adjusted RR) < log crude RR if SRR < crude RR (positive confounding) and E(log adjusted RR) > log crude RR if SRR > crude RR (negative confounding). On the other hand, if U is creating no bias from the outset ($\sigma_{{\text{EOR}}^{-1},\;{\text{DRR}}^{e}}=\sigma_{\text{EOR},\;{\text{DRR}}^{u}}=\sigma {'}_{{\text{EOR}}^{-1},\;{\text{DRR}}^{e}}=\sigma {'}_{\text{EOR},\;{\text{DRR}}^{u}}=0$), there is no need for any further adjustment because the crude RR is already the sought-after SRR. From [4], we see that in this case, adjusting for a PV (even if *f*_PV_ > 0) will not perturb the crude RR.

## Results

### Simulation studies

**Figure 2:**
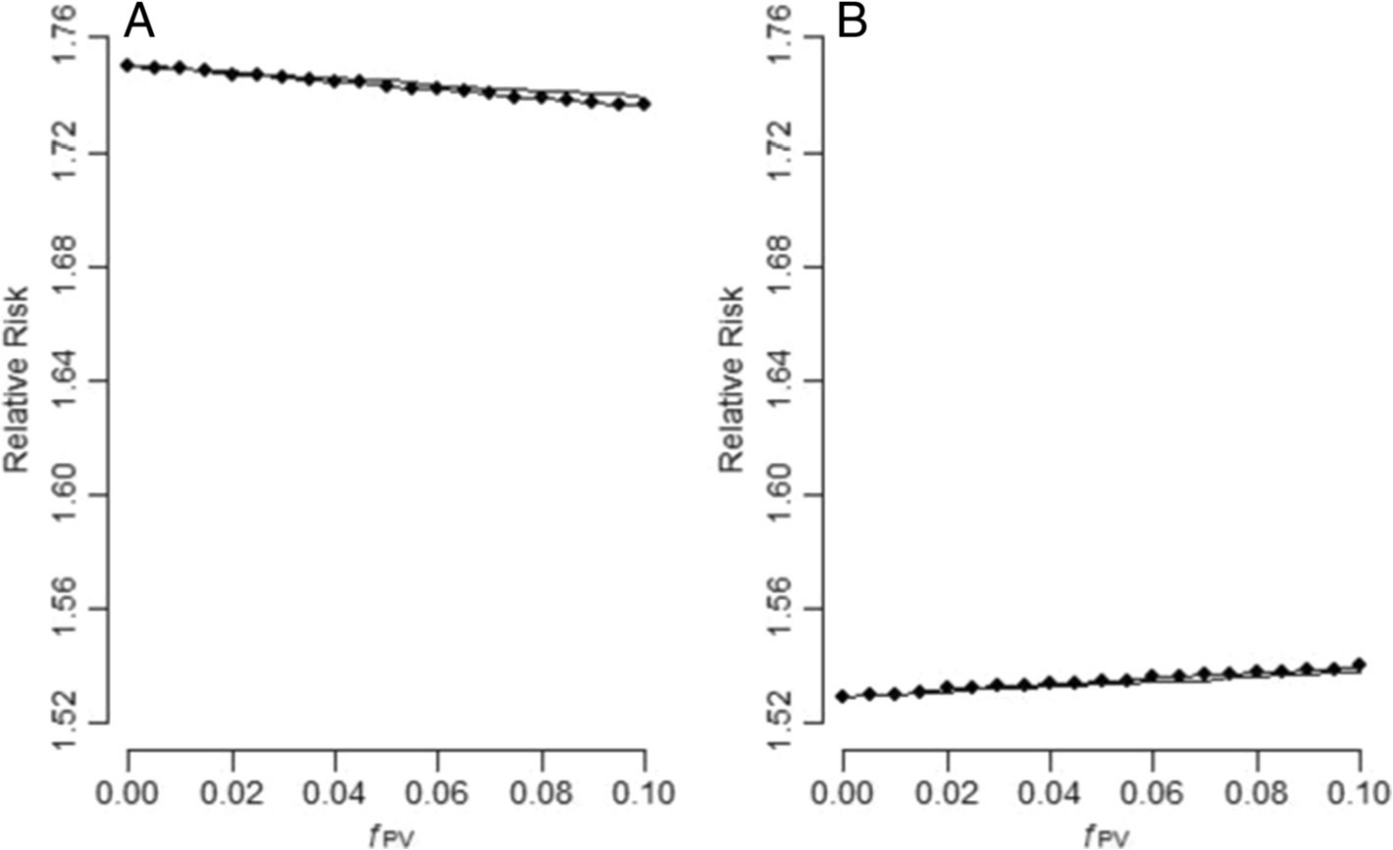
**Effects of the adjustment of a binary perturbation variable for the hypothetical population in Table **1**(A: positive confounding; B: negative confounding; lines with big dot: simulation results; thin lines: Taylor approximation).**

A binary PV for the hypothetical population in Table 1 is simulated. The prevalence of the PV in the four levels of U is assumed to arrive from a beta distribution with *μ*_PV_ = 0.5 and *f*_PV_ = 0.000, 0.005, 0.010, …, 0.100. A total of 100,000 simulations were performed for each scenario. Figure 2 presents the results of the adjustment of the simulated PV for the hypothetical population in Table 1. These data demonstrated that the Taylor approximation formula in [4] agrees quite well with the empirical results (averages of log adjusted RRs in the simulations) and that the adjustments are on average in the right direction for positive confounding (SRR < crude RR, panel A) and negative confounding (SRR > crude RR, panel B). Note that here we are talking about the average; Additional file 2: Tables S1 and S2 present the minimum, Q1, Q3, and maximum of the log adjusted RR from the 100,000 rounds of simulations. Occasionally (though very rarely), adjustment for one strong PV can go in the wrong direction. A strong PV (a measured variable with a very large *f*_PV_) can be considered as a misclassified surrogate for the unmeasured confounder. Ogburn EL, 2012 [6] recently also found that the adjustment for one strong surrogate confounder is not always beneficial. As for the hypothetical population in Table 2 where U is not creating a bias, we found that adjusting for the simulated PV does not perturb the crude RR (Additional file 2: Tables S3–S5).

In situations where the prevalence of PV is distributed as a mixture of beta distributions, the results were basically the same (Additional file 2: Tables S6–S10).

### Perturbation analysis using a panel of perturbation variables

As shown in the previous section, adjusting for an informative PV will produce an adjusted RR that is a little closer, on average, to the unknown SRR than the crude RR is. With only one PV, such a minuscule bias reduction may be unremarkable. However, one can construct a powerful perturbation test (described below) to test whether the study at hand is suffering from the bias of unmeasured factors if one can collect large numbers of PVs. Furthermore, one can perform a perturbation adjustment (also described below) to significantly reduce, if not completely eliminate, that bias.

Note that the PVs to be used can be in any measurement scale. For example, for a categorical variable with a total of five levels, one can create a total of four dummy variables as four separate PVs in the perturbation analysis. A continuous variable counts as one PV, but to extract more information, one can categorize and dummy-code the variable to input more PVs. Alternatively, one can input the variable itself, along with its square, its cube, and so on. Furthermore, interaction terms (product terms) of any subset of already collected PVs by themselves also count as new PVs. It does not matter if some of the PVs, collected or created, are correlated with one another to some degree, as neither the perturbation test nor the perturbation adjustment needs an independence assumption. Additionally, in order to use the method, one does not need to know anything (parameter or function) related to U, such as *L*, *m*_*i*_, *p*_*i*_, *q*_*i*_, $r_{i}^{u}$, $r_{i}^{e}$, $\sigma_{{\text{EOR}}^{-1},\;{\text{DRR}}^{e}}$, $\sigma_{\text{EOR},\;{\text{DRR}}^{u}}$, $\sigma {'}_{{\text{EOR}}^{-1},\;{\text{DRR}}^{e}}$, $\sigma {'}_{\text{EOR},\;{\text{DRR}}^{u}}$, *μ*_PV_, $\sigma_{\text{PV}}^{2}$, or *f*_PV_, etc.

### Perturbation test

Let the panel of PVs be indexed by *k* = 1, 2, …, *m*. The test statistic of the perturbation test is

(5) $$T={\left[\frac{1}{m}\sum_{k=1}^{m}\log\left(\theta_{k}\right)-\log\;\text{crude}\;\text{RR}\right]}^{2},$$

where *θ*_*k*_ is the adjusted RR pertaining to the *k* th PV. From the previous section, we know that under the null hypothesis of no unmeasured confounding, the expected value of the log adjusted RR should equal the log crude RR. Under the alternative, the expected value of the log adjusted RR will be lower (positive confounding) or higher (negative confounding) than the crude RR. Therefore, the value of *T* should tend to be larger under the alternative hypothesis than under the null hypothesis.

Because the PVs may not be independent of one another, the ordinary chi-square distribution may not be appropriate for *T*. Here, we resort to permutation analysis to find a critical value for *T*. To be precise, we fix the vectors (PV_1_, PV_2_, …, PV_*m*_) and shuffle the vectors of (E, D) among the study subjects (or vice versa). Such permutations are to be performed many times, with a *T* value calculated each time. The critical value for a significant level of *α* is then the (1 − *α*) × 100 percentile of these permutated *T* values.

### Perturbation adjustment

To correct the bias of unmeasured factors, one may be tempted to adjust for the whole panel of *m* PVs simultaneously. However, in doing so, one will run into a dimensionality problem. For example, a panel of 20 binary PVs taken together amounts to a super-variable S, with 1,048,676 levels, while in a typical study, the total number of subjects enrolled (*n*) is far less than that number. Therefore, each subject essentially occupies a different level of S, making adjustments of S impossible.

To cope with the problem, a hierarchical clustering algorithm [7] is proposed below to group the subjects into a manageable number of clusters.

1. Start with individual subjects. Let each subject reside in a distinct cluster so that there are as many clusters as subjects.

2. Calculate the distance between any two clusters (for example, the A and the B clusters): $D^{A,B}=\sqrt{\frac{1}{m}\sum_{k=1}^{m}{\left({\text{PV}}_{k}^{A}-{\text{PV}}_{k}^{B}\right)}^{2}},$ where ${\text{PV}}_{k}^{A}$ (${\text{PV}}_{k}^{B}$) is the *k* th PV of the subject in the A(B) cluster.

3. The two clusters (for example, the C and the D clusters) with the smallest distance between them are merged into one cluster (call this the CD cluster).

4. The distance between the newly formed cluster and any other cluster (for example, the E cluster) is calculated as *D*^CD,E^ = max(*D*^C,E^, *D*^D,E^), according to the complete-linkage criterion [7].

5. Repeat Steps 3 and 4 until there are at least a prespecified number of subjects (*n*_*c*_, for example *n*_*c*_ = 20) in each cluster.

Treating these clusters as different levels of the panel of PVs, we then use formula [3] to calculate an adjusted RR. Note that we assume that U itself does not contain too many levels beyond what the sample size of a study can handle, i.e., we assume $L<\frac{n}{n_{c}}$.

## Results

### Simulation studies

To study the performances of the perturbation test and adjustment, a panel of PVs for the hypothetical population in Table 1 was simulated. As before, the prevalences of the PVs in the four levels of U were assumed to arrive from the beta distributions. The mean prevalences (across the four levels of U), $\mu_{{\text{PV}}_{1}},\mu_{{\text{PV}}_{2}},\ldots ,\mu_{{\text{PV}}_{m}}$, were assumed to arrive from a U(0.05,0.95) distribution. The variance fractions, *f*_PV_ s, were assumed to be constant for the panel of PVs and are examined for *f*_PV_ = 0.05 and 0.025. A total of 200 subjects (*n* = 200) were randomly sampled from this population. For a given subject, the values of his/her PV_1_, PV_2_, …, PV_*m*_ were assumed to be independent of one another and were generated from *m* Bernoulli distributions according to the prevalence values of their U levels, without regard to their E and D statuses. One thousand simulations were performed for each scenario. The index of operating characteristic was used to measure the performance of the perturbation test. The operating characteristic of a test is its statistical power averaged over a U(0,1)-distributed *α* -level; it is a value between 0.5 (no power at all) and 1.0 (highest power possible).

**Figure 3:**
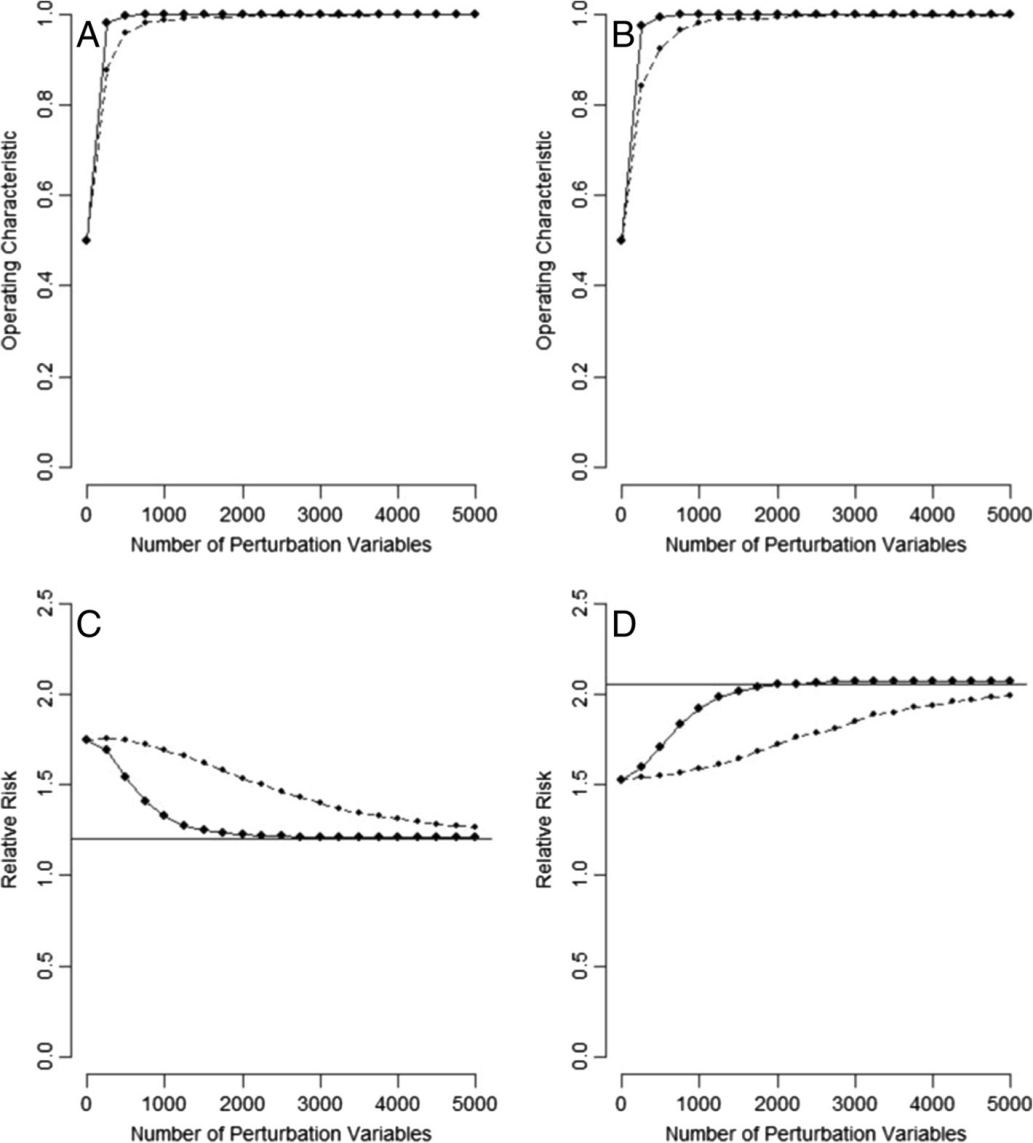
**Results of the perturbation analysis for the hypothetical population in Table **1**(A: perturbation test for positive confounding; B: perturbation test for negative confounding; C: perturbation adjustment for positive confounding; D: perturbation adjustment for negative confounding; solid lines: *f***_**PV**_** = 0.05; dotted lines: *f***_**PV**_** = 0.025; horizontal lines: standardized relative risks).**

Figure 3 presents the simulation results for the hypothetical population in Table 1. As the number of PVs increased, the operating characteristic of the perturbation test increased for detecting hidden positive confounding (panel A) or negative confounding (panel B). Collecting a few hundred PVs for *f*_PV_ = 0.05 (solid lines) or slightly more PVs for *f*_PV_ = 0.025 (dotted lines), allowed hidden confounding to be consistently detected (operating characteristic tending towards 1.0). As for the results of the perturbation adjustment, the adjustments were in the right directions (panel C: positive confounding; panel D: negative confounding). As the number of PVs increased, the adjusted RRs gradually tended to become the respective SRRs (horizontal lines). With a few thousand PVs for *f*_PV_ = 0.05 (solid lines), the bias of U could be removed almost completely (adjusted RR ≈ SRR). For less informative PVs for *f*_PV_ = 0.025 (dotted lines), greater numbers needed to be collected.

When the prevalences of PVs were distributed as a mixture of beta distributions, the results were basically the same (Additional file 3: Figure S1). Additionally, for the situation where the values of PV_1_, PV_2_, …, PV_*m*_ within a subject were dependent, and for the situation where the panel of PVs contained a certain proportion of pure noise (PVs that are not associated with U: *f*_PV_ = 0), definite detection of bias and/or complete removal of bias were also possible, if with an even larger panel of PVs (Additional file 3: Figures S2 and S3).

**Figure 4:**
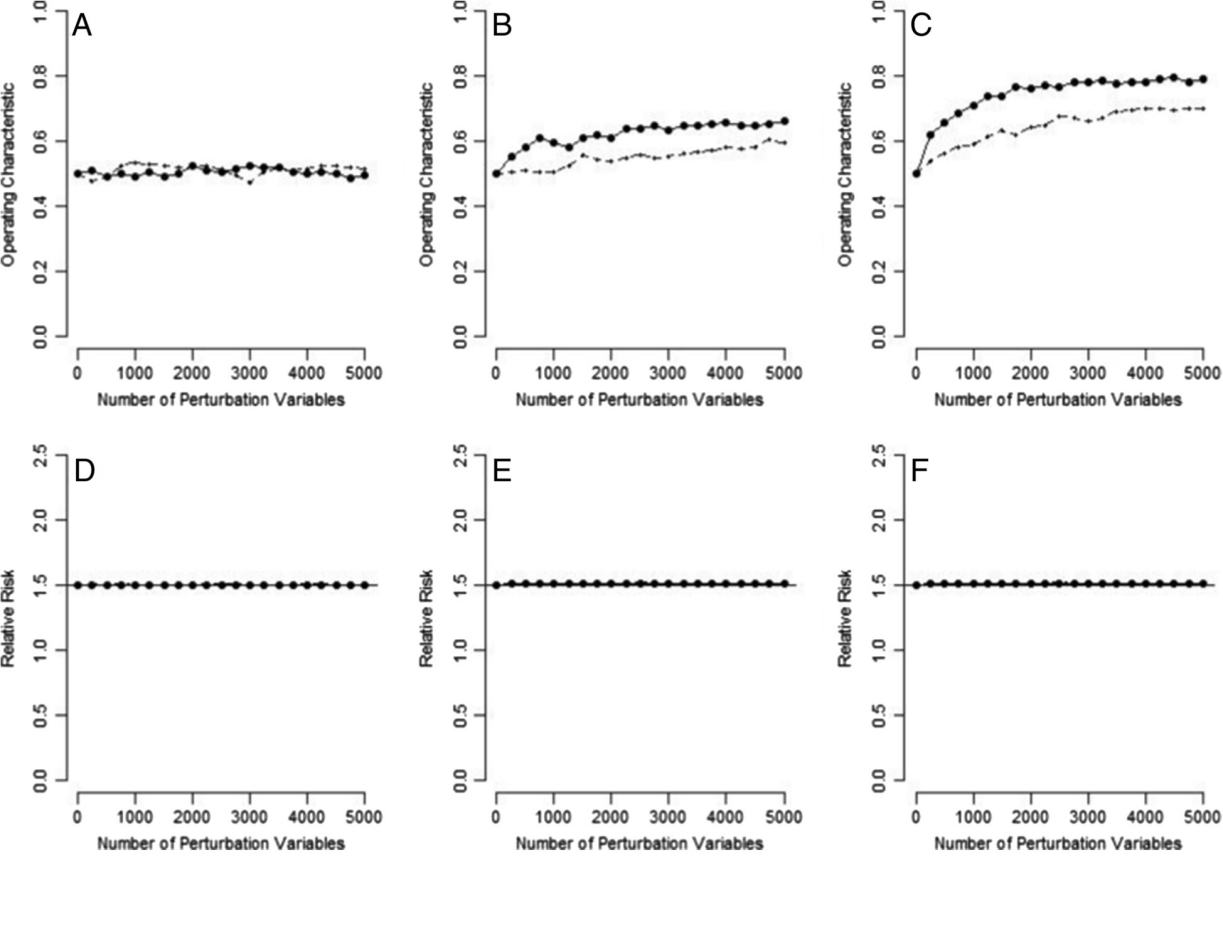
**Results of the perturbation analysis for the hypothetical population in Table **2**(A: perturbation test when the unmeasured is associated with neither exposure nor disease; B: perturbation test when the unmeasured is not associated with exposure but is associated with disease; C: perturbation test when the unmeasured is not associated with disease but is associated with exposure; D: perturbation adjustment when the unmeasured is associated with neither exposure nor disease; E: perturbation adjustment when the unmeasured is not associated with exposure but is associated with disease; F: perturbation adjustment when the unmeasured is not associated with disease but is associated with exposure; solid lines: *f***_**PV**_** = 0.05; dotted lines: *f***_**PV**_** = 0.025; horizontal lines: standardized relative risks).**

Figure 4 presents the simulation results for the hypothetical population in Table 2, where U is not creating bias. When U was associated with neither E nor D, the perturbation test had an operating characteristic of 0.5, i.e., it maintained the correct type I error rate (panel A), and the perturbation adjustment did not perturb the crude RR (crude RR = SRR, in this situation; panel D), irrespective of how many PVs were used. If many PVs were used, the perturbation test had some power (operating characteristic > 0.5) to detect a situation where U was not associated with E, but was associated with D (see panel B) and where U was not associated with D, but was associated with E (see panel E). Even with such sensitivity, the perturbation adjustments correctly stayed at their respective SRR values (the crude RRs themselves), irrespective of how many PVs were used (panels E and F).

## Discussion

We will now comment on why our perturbation analysis using a panel of PVs should work. The perturbation test proposed in this paper centers on the fact that adjusting for an informative PV (a variable associated with U) will produce a value that is on average larger or lower than the crude RR as necessary to be closer to the unknown SRR. This is true irrespective of whether the association between U and PV is positive or negative. Because the adjustments all point in the same direction, we can calculate a test statistic, as in [5], without worrying that the effects of the positively and negatively associated PVs are being cancelled out. Notably, the perturbation adjustment proposed in this paper is based on distances in high dimension. Hall P, 2005 [8] and [9] studied the geometric properties of high-dimension and low-sample-size data. They showed that under very mild conditions, as the dimension (the number of PVs) approaches infinity, the distance between any two subjects in the same group (at the same level of U) will converge to a certain value, while the distance between any two subjects in different groups (at different levels of U) will converge to another (larger) value. Therefore, by calculating pair-wise distances in sufficiently high dimension, the group memberships of the study subjects can be resolved, and U can be reconstructed almost perfectly.

**Figure 5:**
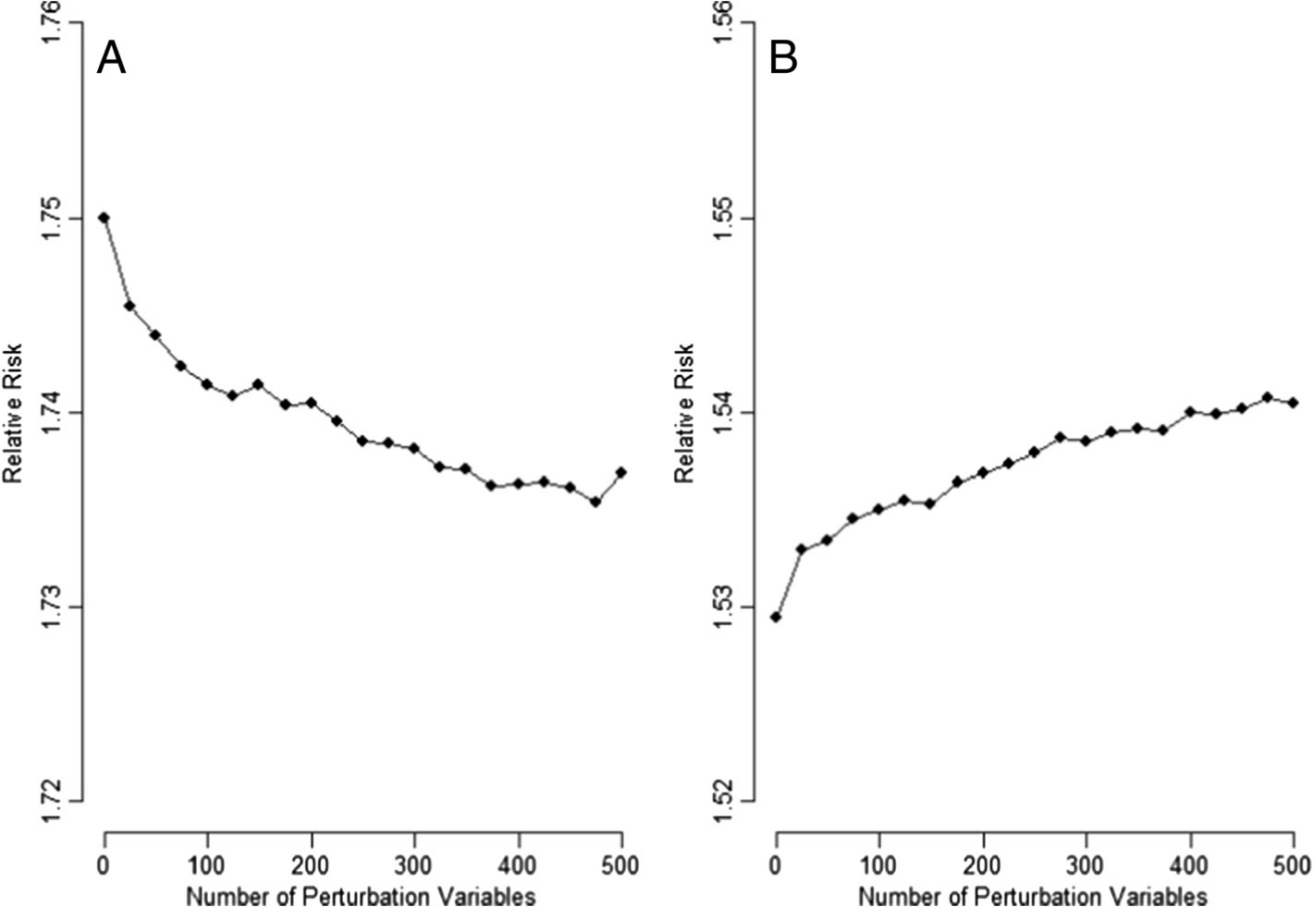
**Perturbation diagnostics for a hypothetical data (*n* = 200) taken from Table **1**(A: perturbation adjustment for positive confounding; B: perturbation adjustment for negative confounding). The perturbation variables have an *f***_**PV **_**of 0.025 and are dependent of one another through a first-order Markov chain with an odds ratio of 10.0 between successive perturbation variables. Bootstrap was done for a total of 10000 times.**

**Figure 6:**
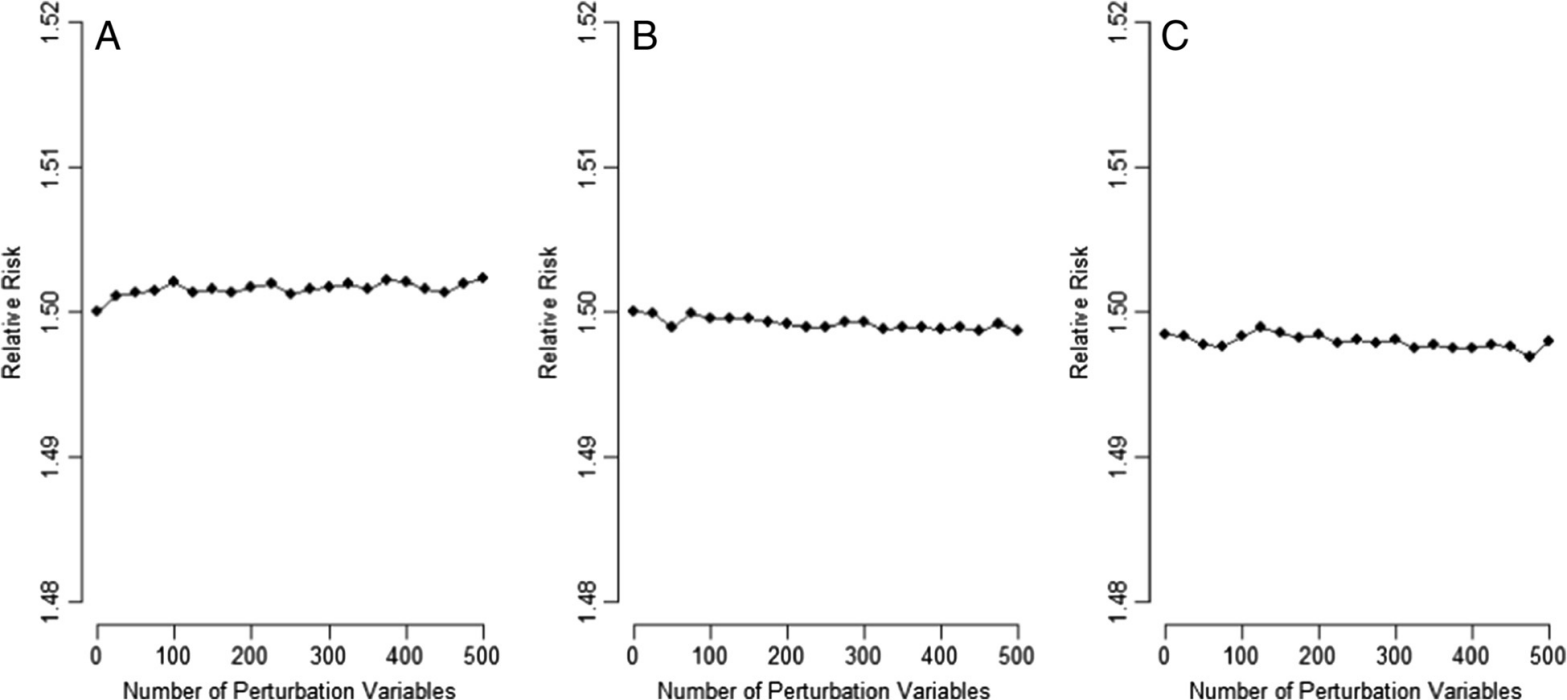
**Perturbation diagnostics for a hypothetical data (*n* = 200) taken from Table **2** (A: perturbation adjustment when the unmeasured is associated with neither exposure nor disease; B: perturbation adjustment when the unmeasured is not associated with exposure but is associated with disease; C: perturbation adjustment when the unmeasured is not associated with disease but is associated with exposure).** The perturbation variables have an *f*_PV_ of 0.025 and are dependent of one another through a first-order Markov chain with an odds ratio of 10.0 between successive perturbation variables. Bootstrap was done for a total of 10000 times.

If practicality issues or cost constraints prevent expanding the panel of available PVs to the thousands or more, one can still make good use of the few hundred PVs in one’s own study (say, a total of 500) for perturbation diagnostics. To be precise, perturbation adjustments can be run using bootstrapped samples (sampling with replacement) of these 500 PVs repeatedly a set number of times (e.g., 10000). The bootstrapped means of the adjusted RRs can be plotted against the number of PVs used. Figures 5 and 6 are hypothetical data from 200 subjects taken from Tables 1 and 2, respectively. The PVs are assumed to be relatively weak (*f*_PV_ = 0.025) and are dependent of one another through a first-order Markov chain with an odds ratio of 10.0 between successive PVs. The trend in the figure is indicative of unmeasured confounding; the direction of the trend (decreasing in Figure 5A; increasing in Figure 5B) also reveals the sign of the bias, while the flat line suggests the absence of confounding (Figure 6A–C).

**Figure 7:**
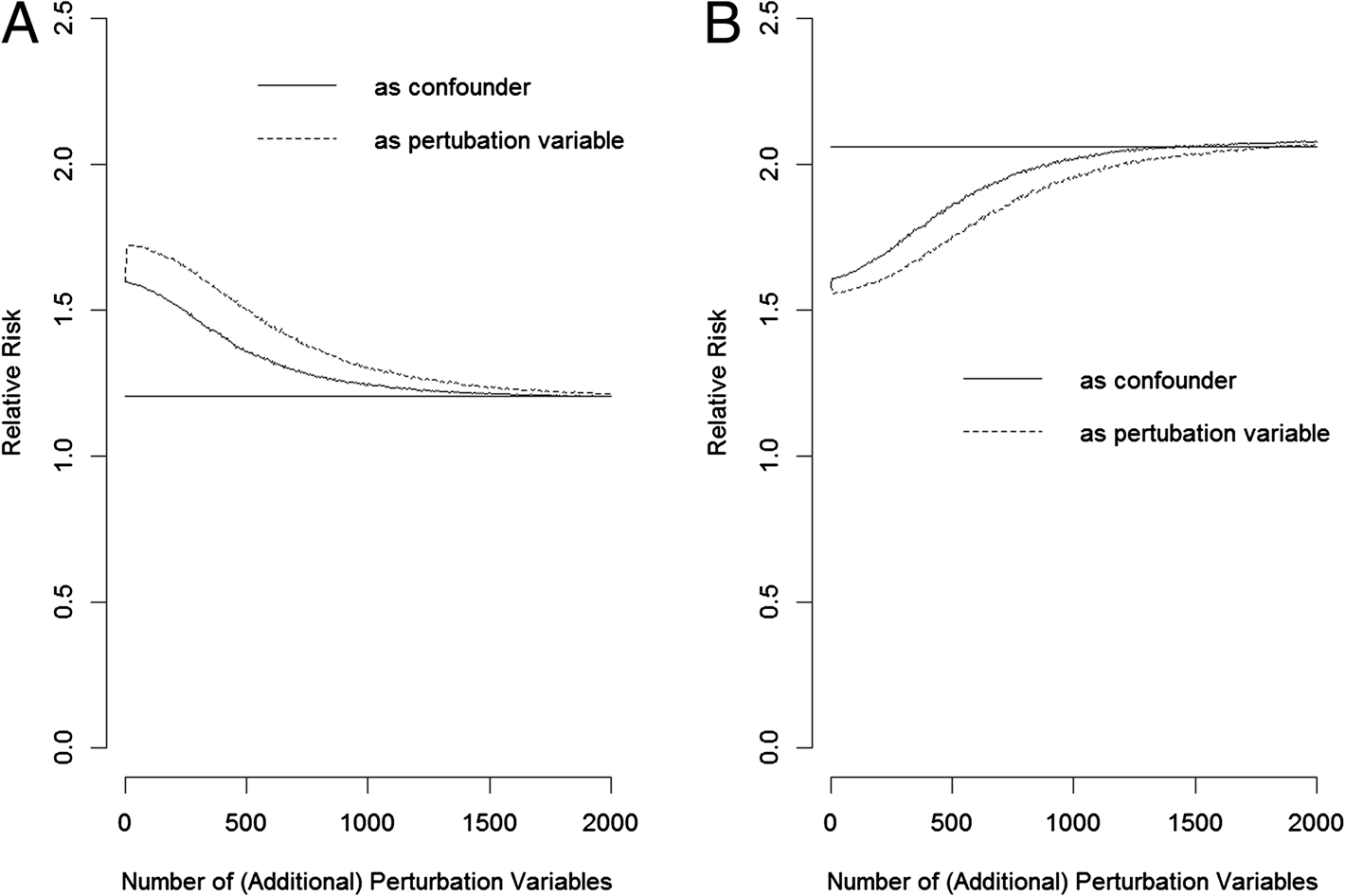
**Perturbation adjustment for the hypothetical population in Table **1**assuming that U is a composite of a measured confounder and a true unknown (A: positive confounding; B: negative confounding).** The measured confounder is treated as a confounder (solid lines), or as a perturbation variable (dotted lines). The (additional) perturbation variables have an *f*_PV_ of 0.05.

To accommodate measured confounders and to further adjust for residual bias, one can perform the same clustering algorithm on the panel of collected PVs as described in this paper, but separately, for each level delineated by the measured confounders. The final adjustment should then be performed with respect to the total resulting clusters. Assuming that the U in Table 1 is actually a composite of a measured confounder (MC) and a true unknown (TU), both being binary variables with (MC, TU) = (1,1) for U = 1, (0,1) for U = 2, (1,0) for U = 3, and (0,0) for U = 4, respectively. Figure 7 shows that treating an MC in this way (as a confounder rather than as an ordinary PV) will speed up the convergence to the true values (compare the solid lines in Figures 7A and 7B with those in Figures 3C and 3D). On the other hand, if a researcher mistakes the MC as a PV (a variable that is associated with E and D only through TU) and treats it as such, we see in Figure 7 (dotted lines) that upon addition of a few more true PVs, the effect of the MC is diluted, and the perturbation adjustment goes in the wrong direction. However, upon addition of more and more PVs, the perturbation adjustment can right itself and then converge to the true values, albeit more slowly than when the MC is correctly specified as a confounder.

The proposed method relies on collecting as many PVs as possible. This is in contrast to other approaches dealing with unmeasured confounding, such as the methods of negative control [10,11], the instrumental variable [12,13], and the latent variable [14], where only one or a few variables are considered. The method is also completely data-driven such that a researcher simply lets the data (consisting of E, D, and a panel of PVs) speak for themselves. This is in contrast to a sensitivity analysis of unmeasured confounding where one needs to specify the sensitivity parameters or assume distributions for them [1,15,16].

There is much work to be performed in order to further validate the proposed method. First, this paper is only a proof-of-concept study. Further studies are needed to test the methodology with real data. Second, additional work is needed to design an optimal coding scheme to extract maximum information from categorical/continuous PVs and a weighting system to optimally combine the many different PVs in the panel in order to maximize the efficiency of the perturbation analysis. Third, the method is currently discussed only on the SRR using the whole population as the target. It will be worthwhile to develop the corresponding methodology for an SRR with the exposed, unexposed, or completely external population as the target. Finally, casting the present method in a proper regression framework should prove useful for accommodating more than two exposures and other confounders that are measured in the study.

## Conclusions

In summary, this study shows that, as the number of PVs increases, the power of the perturbation test increases (progressively up to nearly 100%) and the bias after the perturbation adjustment decreases (progressively down to nearly 0%). Such a data-mining approach is recommended for use in detecting and correcting the biases of unmeasured factors in observation studies.

## Abbreviations

CRR: Confounding risk ratio; DRR: Disease risk ratio; EOR: Exposure odds ratio; PV: Perturbation variable; RR: Relative risk; SRR: Standardized relative risk.

## Competing interests

The author declares that he has no competing interests.

## Author’s contributions

WCL developed the methods, carried out the simulations, and drafted the manuscript. He read and approved the final manuscript.

## Pre-publication history

The pre-publication history for this paper can be accessed here:

http://www.biomedcentral.com/1471-2288/14/18/prepub

## Supplementary Material

Additional file 1 **Supplementary Appendices 1-2.** Derivations of mathematical formulas.
Click here for file

Additional file 2: Tables S1-S10 Additional results of the adjustment of one perturbation variable for the hypothetical population in Tables 1-2.
Click here for file

Additional file 3: Figures S1-S3 Additional results of the perturbation analysis for the hypothetical population in Table 1. Click here for file
