# Supplementary material for: Detecting and correcting the bias of unmeasured factors using perturbation analysis: a data-mining approach
Source: BMC Med Res Methodol. 2014 Feb 5;14:18. doi: 10.1186/1471-2288-14-18 (PMC3925987; doi:10.1186/1471-2288-14-18)
Supplement: Additional file 1 — Supplementary Appendices 1-2. Derivations of mathematical formulas. [file 1471-2288-14-18-S1.doc]

Supplementary Appendix 1.

Using the collapsed population (over U) as the reference, in the $i$th level of U, we first define the exposure odds ratio as $\text{EOR}_{i}=\frac{q_{i}}{\bar{q}}$, the disease risk ratio for the unexposed, $\text{DRR}_{i}^{u}=\frac{r_{i}^{u}}{\bar{r}^{u}}$, and the disease risk ratio for the exposed, $\text{DRR}_{i}^{e}=\frac{r_{i}^{e}}{\bar{r}^{e}}$, respectively. The weighted covariances are then calculated as $\sigma_{\text{EOR},\text{ DRR}^{u}}=\frac{\sum_{i} m_{i}(1-p_{i})(\text{EOR}_{i}-1)(\text{DRR}_{i}^{u}-1)}{\sum_{i} m_{i}(1-p_{i})}$ and $\sigma_{\text{EOR}^{-1},\text{ DRR}^{e}}=\frac{\sum_{i} m_{i}p_{i}(\text{EOR}_{i}^{-1}-1)(\text{DRR}_{i}^{e}-1)}{\sum_{i} m_{i}p_{i}},$ respectively. Using simple algebra, we show that

$$\begin{matrix} \text{CRR}=\frac{\text{crude RR}}{\text{SRR}} \\ \text{ }=\left[\frac{\sum_{i} m_{i}p_{i}r_{i}^{e}}{\sum_{i} m_{i}p_{i}}\right]\times\left[\frac{\sum_{i} m_{i}(1-p_{i})r_{i}^{u}}{\sum_{i} m_{i}(1-p_{i})}\right]^{-1}\times\left[\frac{\sum_{i} m_{i}p_{i}r_{i}^{e}+\sum_{i} m_{i}(1-p_{i})r_{i}^{e}}{\sum_{i} m_{i}(1-p_{i})r_{i}^{u}+\sum_{i} m_{i}p_{i}r_{i}^{u}}\right]^{-1} \\ \text{ }=\left\{\sum_{i} m_{i}(1-p_{i})+\left[\sum_{i} m_{i}p_{i}\right]\times\frac{\left[\sum_{i} m_{i}(1-p_{i})\right]\times\left[\sum_{i} m_{i}p_{i}r_{i}^{u}\right]}{\text{ }\left[\sum_{i} m_{i}p_{i}\right]\times\left[\sum_{i} m_{i}(1-p_{i})r_{i}^{u}\right]\text{ }}\right\} \\ \text{ }\times\left\{\sum_{i} m_{i}p_{i}+\left[\sum_{i} m_{i}(1-p_{i})\right]\times\frac{\left[\sum_{i} m_{i}p_{i}\right]\times\left[\sum_{i} m_{i}(1-p_{i})r_{i}^{e}\right]}{\left[\sum_{i} m_{i}(1-p_{i})\right]\times\left[\sum_{i} m_{i}p_{i}r_{i}^{e}\right]}\right\}^{-1} \\ \text{ }=\left\{\sum_{i} m_{i}(1-p_{i})+\left[\sum_{i} m_{i}p_{i}\right]\times\left[1+\sigma_{\text{EOR},\text{ DRR}^{u}}\right]\right\} \\ \text{ }\times\left\{\sum_{i} m_{i}p_{i}+\left[\sum_{i} m_{i}(1-p_{i})\right]\times\left[1+\sigma_{\text{EOR}^{-1},\text{ DRR}^{e}}\right]\right\}^{-1} \\ \text{ }=\frac{1+\bar{p}\times\sigma_{\text{EOR},\text{ DRR}^{u}}}{1+(1-\bar{p})\times\sigma_{\text{EOR}^{-1},\text{ DRR}^{e}}}. \end{matrix}$$

Supplementary Appendix 2.

Let $x_{i}$ denote the prevalence of the binary PV at the $i$th level of U. We first express the log adjusted RR as a function of $x_{1},...,x_{L}$:

$$\begin{matrix} \text{log adjusted RR}=g(x_{1},...,x_{L}) \\ \text{ }=\log\left\{\left[\sum_{i} m_{i}x_{i}\right]\times\frac{\sum_{i} m_{i}p_{i}x_{i}r_{i}^{e}}{\sum_{i} m_{i}p_{i}x_{i}}+\left[\sum_{i} m_{i}(1-x_{i})\right]\times\frac{\sum_{i} m_{i}p_{i}(1-x_{i})r_{i}^{e}}{\sum_{i} m_{i}p_{i}(1-x_{i})}\right\} \\ \text{ }-\log\left\{\left[\sum_{i} m_{i}x_{i}\right]\times\frac{\sum_{i} m_{i}p_{i}x_{i}r_{i}^{u}}{\sum_{i} m_{i}p_{i}x_{i}}+\left[\sum_{i} m_{i}(1-x_{i})\right]\times\frac{\sum_{i} m_{i}p_{i}(1-x_{i})r_{i}^{u}}{\sum_{i} m_{i}p_{i}(1-x_{i})}\right\}. \end{matrix}$$

Next, we approximate $g(x_{1},...,x_{L})$ using Taylor series to second order about the point $(\mu_{\text{PV}},...,\mu_{\text{PV}})$:

$$\begin{matrix} g(x_{1},...,x_{L})\approx g(\mu_{\text{PV}},...,\mu_{\text{PV}}) \\ \text{ }+\sum_{i=1}^{L} (x_{i}-\mu_{\text{PV}})\times g_{x_{i}}(\mu_{\text{PV}},...,\mu_{\text{PV}}) \\ \text{ }+\frac{1}{2}\times\sum_{i=1}^{L} \sum_{j=1}^{L} (x_{i}-\mu_{\text{PV}})\times(x_{j}-\mu_{\text{PV}})\times g_{x_{i}x_{j}}(\mu_{\text{PV}},...,\mu_{\text{PV}}), \end{matrix}$$

where the subscript of $g(x_{1},...,x_{L})$ denote the respective partial derivatives. Assume that the $x_{i}$’s are distributed independently and identically, with a common mean of $\mu_{\text{PV}}$ and a common variance of $\sigma_{\text{PV}}^{2}$. With some algebra, it can be shown that

$$\begin{matrix} \text{E}(\text{log adjusted RR})=\text{E}[g(x_{1},...,x_{L})] \\ \text{ }\approx g(\mu_{\text{PV}},...,\mu_{\text{PV}})+\frac{1}{2}\times\sum_{i=1}^{L} \sigma_{\text{PV}}^{2}\times g_{x_{i}x_{i}}(\mu_{\text{PV}},...,\mu_{\text{PV}}) \\ \text{ }=\text{log crude RR}+\left(a\times\sigma'_{\text{EOR}^{-1},\text{ DRR}^{e}}-b\times\sigma'_{\text{EOR},\text{ DRR}^{u}}\right)\times f_{\text{PV}}, \end{matrix}$$

where $\sigma'_{\text{EOR}^{-1},\text{ DRR}^{e}}=\frac{\sum_{i} (m_{i}p_{i})^{2}(\text{EOR}_{i}^{-1}-1)(\text{DRR}_{i}^{e}-1)}{\sum_{i} (m_{i}p_{i})^{2}}$, $\sigma'_{\text{EOR},\text{ DRR}^{u}}=\frac{\sum_{i} m_{i}^{2}(1-p_{i})^{2}(\text{EOR}_{i}-1)(\text{DRR}_{i}^{u}-1)}{\sum_{i} m_{i}^{2}(1-p_{i})^{2}}$, $f_{\text{PV}}=\frac{\sigma_{\text{PV}}^{2}}{\mu_{\text{PV}}\times(1-\mu_{\text{PV}})}$, $a=(1-\bar{p})\times\left[\frac{1}{L}+\sum_{i} \left(\frac{m_{i}p_{i}}{\sum_{j} m_{j}p_{j}}-\frac{1}{L}\right)^{2}\right]$, and $b=\bar{p}\times\left[\frac{1}{L}+\sum_{i} \left(\frac{m_{i}(1-p_{i})}{\sum_{j} m_{j}(1-p_{j})}-\frac{1}{L}\right)^{2}\right].$ ($\text{EOR}_{i}$, $\text{DRR}_{i}^{u}$, and $\text{DRR}_{i}^{e}$ are defined in Supplementary Appendix 1.)
