# Supplementary material for: Detecting and correcting the bias of unmeasured factors using perturbation analysis: a data-mining approach
Source: BMC Med Res Methodol. 2014 Feb 5;14:18. doi: 10.1186/1471-2288-14-18 (PMC3925987; doi:10.1186/1471-2288-14-18)
Supplement: Additional file 2: Tables S1-S10 — Additional results of the adjustment of one perturbation variable for the hypothetical population in Tables 1-2. [file 1471-2288-14-18-S2.doc]

Table S1. Results of the adjustment of one perturbation variable for the hypothetical population in Table 1 with positive confounding bias. The prevalence of the perturbation variable in the four levels of the unmeasured is assumed to arrive from a beta distribution.

____________________________________________________________________________

Adjusted RR Adjusted RRs from 100,000 simulations

$f_{\text{PV}}$ from equation [4] __________________________________________________

in text Minimum Q1 Mean Q3 Maximum

____________________________________________________________________________

0.000 1.750 1.750 1.750 1.750 1.750 1.750

0.005 1.750 1.737 1.749 1.749 1.750 1.750

0.010 1.749 1.719 1.748 1.749 1.750 1.751

0.015 1.748 1.710 1.747 1.748 1.750 1.751

0.020 1.748 1.703 1.746 1.747 1.750 1.752

0.025 1.747 1.694 1.745 1.747 1.750 1.752

0.030 1.747 1.673 1.744 1.746 1.750 1.752

0.035 1.746 1.673 1.743 1.745 1.750 1.752

0.040 1.746 1.658 1.743 1.744 1.750 1.752

0.045 1.745 1.638 1.742 1.744 1.750 1.753

0.050 1.745 1.614 1.740 1.743 1.749 1.752

0.055 1.744 1.622 1.739 1.742 1.749 1.753

0.060 1.743 1.618 1.739 1.742 1.749 1.753

0.065 1.743 1.588 1.737 1.741 1.749 1.753

0.070 1.742 1.595 1.736 1.740 1.749 1.753

0.075 1.742 1.578 1.735 1.739 1.749 1.754

0.080 1.741 1.565 1.734 1.739 1.749 1.753

0.085 1.741 1.563 1.733 1.738 1.749 1.754

0.090 1.740 1.546 1.732 1.737 1.749 1.754

0.095 1.740 1.551 1.731 1.736 1.749 1.754

0.100 1.739 1.546 1.730 1.736 1.749 1.754

____________________________________________________________________________

Table S2. Results of the adjustment of one perturbation variable for the hypothetical population in Table 1 with negative confounding bias. The prevalence of the perturbation variable in the four levels of the unmeasured is assumed to arrive from a beta distribution.

____________________________________________________________________________

Adjusted RR Adjusted RRs from 100,000 simulations

$f_{\text{PV}}$ from equation [4] __________________________________________________

in text Minimum Q1 Mean Q3 Maximum

____________________________________________________________________________

0.000 1.529 1.529 1.529 1.529 1.529 1.529

0.005 1.530 1.529 1.529 1.530 1.530 1.539

0.010 1.530 1.529 1.530 1.530 1.531 1.550

0.015 1.531 1.529 1.530 1.531 1.531 1.557

0.020 1.531 1.529 1.530 1.532 1.532 1.577

0.025 1.532 1.529 1.530 1.532 1.533 1.577

0.030 1.532 1.529 1.530 1.533 1.534 1.585

0.035 1.532 1.529 1.530 1.533 1.534 1.607

0.040 1.533 1.529 1.530 1.534 1.535 1.594

0.045 1.533 1.529 1.530 1.534 1.536 1.615

0.050 1.534 1.529 1.530 1.535 1.536 1.636

0.055 1.534 1.529 1.530 1.535 1.537 1.627

0.060 1.535 1.529 1.530 1.536 1.538 1.658

0.065 1.535 1.529 1.530 1.536 1.539 1.652

0.070 1.535 1.529 1.530 1.537 1.539 1.666

0.075 1.536 1.529 1.530 1.537 1.540 1.667

0.080 1.536 1.529 1.530 1.538 1.541 1.662

0.085 1.537 1.529 1.530 1.538 1.541 1.687

0.090 1.537 1.529 1.530 1.539 1.542 1.695

0.095 1.538 1.529 1.530 1.539 1.543 1.695

0.100 1.538 1.529 1.531 1.540 1.544 1.710

____________________________________________________________________________

Table S3. Results of the adjustment of one perturbation variable for the hypothetical population in Table 2, when the unmeasured is associated with neither exposure nor disease. The prevalence of the perturbation variable in the four levels of the unmeasured is assumed to arrive from a beta distribution.

____________________________________________________________________________

Adjusted RR Adjusted RRs from 100,000 simulations

$f_{\text{PV}}$ from equation [4] __________________________________________________

in text Minimum Q1 Mean Q3 Maximum

____________________________________________________________________________

0.000 1.500 1.500 1.500 1.500 1.500 1.500

0.005 1.500 1.500 1.500 1.500 1.500 1.500

0.010 1.500 1.500 1.500 1.500 1.500 1.500

0.015 1.500 1.500 1.500 1.500 1.500 1.500

0.020 1.500 1.500 1.500 1.500 1.500 1.500

0.025 1.500 1.500 1.500 1.500 1.500 1.500

0.030 1.500 1.500 1.500 1.500 1.500 1.500

0.035 1.500 1.500 1.500 1.500 1.500 1.500

0.040 1.500 1.500 1.500 1.500 1.500 1.500

0.045 1.500 1.500 1.500 1.500 1.500 1.500

0.050 1.500 1.500 1.500 1.500 1.500 1.500

0.055 1.500 1.500 1.500 1.500 1.500 1.500

0.060 1.500 1.500 1.500 1.500 1.500 1.500

0.065 1.500 1.500 1.500 1.500 1.500 1.500

0.070 1.500 1.500 1.500 1.500 1.500 1.500

0.075 1.500 1.500 1.500 1.500 1.500 1.500

0.080 1.500 1.500 1.500 1.500 1.500 1.500

0.085 1.500 1.500 1.500 1.500 1.500 1.500

0.090 1.500 1.500 1.500 1.500 1.500 1.500

0.095 1.500 1.500 1.500 1.500 1.500 1.500

0.100 1.500 1.500 1.500 1.500 1.500 1.500

____________________________________________________________________________

Table S4. Results of the adjustment of one perturbation variable for the hypothetical population in Table 2, when the unmeasured is not associated with exposure but is associated with disease. The prevalence of the perturbation variable in the four levels of the unmeasured is assumed to arrive from a beta distribution.

____________________________________________________________________________

Adjusted RR Adjusted RRs from 100,000 simulations

$f_{\text{PV}}$ from equation [4] __________________________________________________

in text Minimum Q1 Mean Q3 Maximum

____________________________________________________________________________

0.000 1.500 1.500 1.500 1.500 1.500 1.500

0.005 1.500 1.500 1.500 1.500 1.500 1.500

0.010 1.500 1.500 1.500 1.500 1.500 1.500

0.015 1.500 1.500 1.500 1.500 1.500 1.500

0.020 1.500 1.500 1.500 1.500 1.500 1.500

0.025 1.500 1.500 1.500 1.500 1.500 1.500

0.030 1.500 1.500 1.500 1.500 1.500 1.500

0.035 1.500 1.500 1.500 1.500 1.500 1.500

0.040 1.500 1.500 1.500 1.500 1.500 1.500

0.045 1.500 1.500 1.500 1.500 1.500 1.500

0.050 1.500 1.500 1.500 1.500 1.500 1.500

0.055 1.500 1.500 1.500 1.500 1.500 1.500

0.060 1.500 1.500 1.500 1.500 1.500 1.500

0.065 1.500 1.500 1.500 1.500 1.500 1.500

0.070 1.500 1.500 1.500 1.500 1.500 1.500

0.075 1.500 1.500 1.500 1.500 1.500 1.500

0.080 1.500 1.500 1.500 1.500 1.500 1.500

0.085 1.500 1.500 1.500 1.500 1.500 1.500

0.090 1.500 1.500 1.500 1.500 1.500 1.500

0.095 1.500 1.500 1.500 1.500 1.500 1.500

0.100 1.500 1.500 1.500 1.500 1.500 1.500

____________________________________________________________________________

Table S5. Results of the adjustment of one perturbation variable for the hypothetical population in Table 2, when the unmeasured is not associated with disease but is associated with exposure. The prevalence of the perturbation variable in the four levels of the unmeasured is assumed to arrive from a beta distribution.

____________________________________________________________________________

Adjusted RR Adjusted RRs from 100,000 simulations

$f_{\text{PV}}$ from equation [4] __________________________________________________

in text Minimum Q1 Mean Q3 Maximum

____________________________________________________________________________

0.000 1.500 1.500 1.500 1.500 1.500 1.500

0.005 1.500 1.500 1.500 1.500 1.500 1.500

0.010 1.500 1.500 1.500 1.500 1.500 1.500

0.015 1.500 1.500 1.500 1.500 1.500 1.500

0.020 1.500 1.500 1.500 1.500 1.500 1.500

0.025 1.500 1.500 1.500 1.500 1.500 1.500

0.030 1.500 1.500 1.500 1.500 1.500 1.500

0.035 1.500 1.500 1.500 1.500 1.500 1.500

0.040 1.500 1.500 1.500 1.500 1.500 1.500

0.045 1.500 1.500 1.500 1.500 1.500 1.500

0.050 1.500 1.500 1.500 1.500 1.500 1.500

0.055 1.500 1.500 1.500 1.500 1.500 1.500

0.060 1.500 1.500 1.500 1.500 1.500 1.500

0.065 1.500 1.500 1.500 1.500 1.500 1.500

0.070 1.500 1.500 1.500 1.500 1.500 1.500

0.075 1.500 1.500 1.500 1.500 1.500 1.500

0.080 1.500 1.500 1.500 1.500 1.500 1.500

0.085 1.500 1.500 1.500 1.500 1.500 1.500

0.090 1.500 1.500 1.500 1.500 1.500 1.500

0.095 1.500 1.500 1.500 1.500 1.500 1.500

0.100 1.500 1.500 1.500 1.500 1.500 1.500

____________________________________________________________________________

Table S6. Results of the adjustment of one perturbation variable for the hypothetical population in Table 1 with positive confounding bias. The prevalence of the perturbation variable in the four levels of the unmeasured is assumed to arrive from a 50:50 mixture of two beta distributions.

____________________________________________________________________________

Adjusted RR Adjusted RRs from 100,000 simulations

$f_{\text{PV}}$ from equation [4] _______________________________________________

in text Minimum Q1 Mean Q3 Maximum

____________________________________________________________________________

0,0000, 0.0000 1.750 1.750 1.750 1.750 1.750 1.750

0.0025, 0.0075 1.750 1.724 1.749 1.749 1.750 1.750

0.0050, 0.0150 1.749 1.713 1.748 1.749 1.750 1.751

0.0075, 0.0225 1.748 1.692 1.747 1.748 1.750 1.751

0.0100, 0.0300 1.748 1.688 1.747 1.747 1.750 1.752

0.0125, 0.0375 1.747 1.672 1.746 1.747 1.750 1.752

0.0150, 0.0450 1.747 1.669 1.745 1.746 1.750 1.752

0.0175, 0.0525 1.746 1.646 1.744 1.745 1.750 1.752

0.0200, 0.0600 1.746 1.634 1.743 1.744 1.750 1.753

0.0225, 0.0675 1.745 1.622 1.742 1.744 1.750 1.753

0.0250, 0.0750 1.745 1.600 1.741 1.743 1.750 1.754

0.0275, 0.0825 1.744 1.582 1.740 1.742 1.749 1.753

0.0300, 0.0900 1.743 1.581 1.739 1.741 1.749 1.753

0.0325, 0.0975 1.743 1.571 1.738 1.741 1.749 1.753

0.0350, 0.1050 1.742 1.568 1.737 1.740 1.749 1.754

0.0375, 0.1125 1.742 1.565 1.736 1.739 1.749 1.753

0.0400, 0.1200 1.741 1.548 1.735 1.739 1.749 1.755

0.0425, 0.1275 1.741 1.533 1.734 1.738 1.749 1.755

0.0450, 0.1350 1.740 1.525 1.733 1.737 1.749 1.754

0.0475, 0.1425 1.740 1.526 1.732 1.736 1.749 1.755

0.0500, 0.1500 1.739 1.524 1.731 1.736 1.749 1.754

____________________________________________________________________________

Table S7. Results of the adjustment of one perturbation variable for the hypothetical population in Table 1 with negative confounding bias. The prevalence of the perturbation variable in the four levels of the unmeasured is assumed to arrive from a 50:50 mixture of two beta distributions.

____________________________________________________________________________

Adjusted RR Adjusted RRs from 100,000 simulations

$f_{\text{PV}}$ from equation [4] _______________________________________________

in text Minimum Q1 Mean Q3 Maximum

____________________________________________________________________________

0,0000, 0.0000 1.529 1.529 1.529 1.529 1.529 1.529

0.0025, 0.0075 1.530 1.529 1.529 1.530 1.530 1.544

0.0050, 0.0150 1.530 1.529 1.530 1.530 1.531 1.555

0.0075, 0.0225 1.531 1.529 1.530 1.531 1.531 1.568

0.0100, 0.0300 1.531 1.529 1.530 1.531 1.532 1.574

0.0125, 0.0375 1.532 1.529 1.530 1.532 1.533 1.597

0.0150, 0.0450 1.532 1.529 1.530 1.533 1.533 1.615

0.0175, 0.0525 1.532 1.529 1.530 1.533 1.534 1.635

0.0200, 0.0600 1.533 1.529 1.530 1.534 1.535 1.657

0.0225, 0.0675 1.533 1.529 1.530 1.534 1.536 1.645

0.0250, 0.0750 1.534 1.529 1.530 1.535 1.536 1.632

0.0275, 0.0825 1.534 1.529 1.530 1.535 1.537 1.651

0.0300, 0.0900 1.535 1.529 1.530 1.536 1.538 1.654

0.0325, 0.0975 1.535 1.529 1.530 1.536 1.538 1.675

0.0350, 0.1050 1.535 1.529 1.530 1.537 1.539 1.677

0.0375, 0.1125 1.536 1.529 1.530 1.537 1.540 1.700

0.0400, 0.1200 1.536 1.529 1.530 1.538 1.541 1.688

0.0425, 0.1275 1.537 1.529 1.530 1.538 1.541 1.699

0.0450, 0.1350 1.537 1.529 1.530 1.539 1.542 1.723

0.0475, 0.1425 1.538 1.529 1.530 1.540 1.543 1.754

0.0500, 0.1500 1.538 1.529 1.530 1.540 1.544 1.737

____________________________________________________________________________

Table S8. Results of the adjustment of one perturbation variable for the hypothetical population in Table 2, when the unmeasured is associated with neither exposure nor disease. The prevalence of the perturbation variable in the four levels of the unmeasured is assumed to arrive from a 50:50 mixture of two beta distributions.

____________________________________________________________________________

Adjusted RR Adjusted RRs from 100,000 simulations

$f_{\text{PV}}$ from equation [4] _______________________________________________

in text Minimum Q1 Mean Q3 Maximum

____________________________________________________________________________

0,0000, 0.0000 1.500 1.500 1.500 1.500 1.500 1.500

0.0025, 0.0075 1.500 1.500 1.500 1.500 1.500 1.500

0.0050, 0.0150 1.500 1.500 1.500 1.500 1.500 1.500

0.0075, 0.0225 1.500 1.500 1.500 1.500 1.500 1.500

0.0100, 0.0300 1.500 1.500 1.500 1.500 1.500 1.500

0.0125, 0.0375 1.500 1.500 1.500 1.500 1.500 1.500

0.0150, 0.0450 1.500 1.500 1.500 1.500 1.500 1.500

0.0175, 0.0525 1.500 1.500 1.500 1.500 1.500 1.500

0.0200, 0.0600 1.500 1.500 1.500 1.500 1.500 1.500

0.0225, 0.0675 1.500 1.500 1.500 1.500 1.500 1.500

0.0250, 0.0750 1.500 1.500 1.500 1.500 1.500 1.500

0.0275, 0.0825 1.500 1.500 1.500 1.500 1.500 1.500

0.0300, 0.0900 1.500 1.500 1.500 1.500 1.500 1.500

0.0325, 0.0975 1.500 1.500 1.500 1.500 1.500 1.500

0.0350, 0.1050 1.500 1.500 1.500 1.500 1.500 1.500

0.0375, 0.1125 1.500 1.500 1.500 1.500 1.500 1.500

0.0400, 0.1200 1.500 1.500 1.500 1.500 1.500 1.500

0.0425, 0.1275 1.500 1.500 1.500 1.500 1.500 1.500

0.0450, 0.1350 1.500 1.500 1.500 1.500 1.500 1.500

0.0475, 0.1425 1.500 1.500 1.500 1.500 1.500 1.500

0.0500, 0.1500 1.500 1.500 1.500 1.500 1.500 1.500

____________________________________________________________________________

Table S9. Results of the adjustment of one perturbation variable for the hypothetical population in Table 2, when the unmeasured is not associated with exposure but is associated with disease. The prevalence of the perturbation variable in the four levels of the unmeasured is assumed to arrive from a 50:50 mixture of two beta distributions.

____________________________________________________________________________

Adjusted RR Adjusted RRs from 100,000 simulations

$f_{\text{PV}}$ from equation [4] _______________________________________________

in text Minimum Q1 Mean Q3 Maximum

____________________________________________________________________________

0,0000, 0.0000 1.500 1.500 1.500 1.500 1.500 1.500

0.0025, 0.0075 1.500 1.500 1.500 1.500 1.500 1.500

0.0050, 0.0150 1.500 1.500 1.500 1.500 1.500 1.500

0.0075, 0.0225 1.500 1.500 1.500 1.500 1.500 1.500

0.0100, 0.0300 1.500 1.500 1.500 1.500 1.500 1.500

0.0125, 0.0375 1.500 1.500 1.500 1.500 1.500 1.500

0.0150, 0.0450 1.500 1.500 1.500 1.500 1.500 1.500

0.0175, 0.0525 1.500 1.500 1.500 1.500 1.500 1.500

0.0200, 0.0600 1.500 1.500 1.500 1.500 1.500 1.500

0.0225, 0.0675 1.500 1.500 1.500 1.500 1.500 1.500

0.0250, 0.0750 1.500 1.500 1.500 1.500 1.500 1.500

0.0275, 0.0825 1.500 1.500 1.500 1.500 1.500 1.500

0.0300, 0.0900 1.500 1.500 1.500 1.500 1.500 1.500

0.0325, 0.0975 1.500 1.500 1.500 1.500 1.500 1.500

0.0350, 0.1050 1.500 1.500 1.500 1.500 1.500 1.500

0.0375, 0.1125 1.500 1.500 1.500 1.500 1.500 1.500

0.0400, 0.1200 1.500 1.500 1.500 1.500 1.500 1.500

0.0425, 0.1275 1.500 1.500 1.500 1.500 1.500 1.500

0.0450, 0.1350 1.500 1.500 1.500 1.500 1.500 1.500

0.0475, 0.1425 1.500 1.500 1.500 1.500 1.500 1.500

0.0500, 0.1500 1.500 1.500 1.500 1.500 1.500 1.500

____________________________________________________________________________

Table S10. Results of the adjustment of one perturbation variable for the hypothetical population in Table 2, when the unmeasured is not associated with disease but is associated with exposure. The prevalence of the perturbation variable in the four levels of the unmeasured is assumed to arrive from a 50:50 mixture of two beta distributions.

____________________________________________________________________________

Adjusted RR Adjusted RRs from 100,000 simulations

$f_{\text{PV}}$ from equation [4] _______________________________________________

in text Minimum Q1 Mean Q3 Maximum

____________________________________________________________________________

0,0000, 0.0000 1.500 1.500 1.500 1.500 1.500 1.500

0.0025, 0.0075 1.500 1.500 1.500 1.500 1.500 1.500

0.0050, 0.0150 1.500 1.500 1.500 1.500 1.500 1.500

0.0075, 0.0225 1.500 1.500 1.500 1.500 1.500 1.500

0.0100, 0.0300 1.500 1.500 1.500 1.500 1.500 1.500

0.0125, 0.0375 1.500 1.500 1.500 1.500 1.500 1.500

0.0150, 0.0450 1.500 1.500 1.500 1.500 1.500 1.500

0.0175, 0.0525 1.500 1.500 1.500 1.500 1.500 1.500

0.0200, 0.0600 1.500 1.500 1.500 1.500 1.500 1.500

0.0225, 0.0675 1.500 1.500 1.500 1.500 1.500 1.500

0.0250, 0.0750 1.500 1.500 1.500 1.500 1.500 1.500

0.0275, 0.0825 1.500 1.500 1.500 1.500 1.500 1.500

0.0300, 0.0900 1.500 1.500 1.500 1.500 1.500 1.500

0.0325, 0.0975 1.500 1.500 1.500 1.500 1.500 1.500

0.0350, 0.1050 1.500 1.500 1.500 1.500 1.500 1.500

0.0375, 0.1125 1.500 1.500 1.500 1.500 1.500 1.500

0.0400, 0.1200 1.500 1.500 1.500 1.500 1.500 1.500

0.0425, 0.1275 1.500 1.500 1.500 1.500 1.500 1.500

0.0450, 0.1350 1.500 1.500 1.500 1.500 1.500 1.500

0.0475, 0.1425 1.500 1.500 1.500 1.500 1.500 1.500

0.0500, 0.1500 1.500 1.500 1.500 1.500 1.500 1.500

____________________________________________________________________________
