# Supplementary material for: Detecting and correcting the bias of unmeasured factors using perturbation analysis: a data-mining approach
Source: BMC Med Res Methodol. 2014 Feb 5;14:18. doi: 10.1186/1471-2288-14-18 (PMC3925987; doi:10.1186/1471-2288-14-18)
Supplement: Additional file 3: Figures S1-S3 — Additional results of the perturbation analysis for the hypothetical population in Table 1. [file 1471-2288-14-18-S3.doc]

Supplementary Figure Legends

Figure S1. Results of the perturbation analysis for the hypothetical population in Table 1, when the prevalence of perturbation variables is distributed as a mixture of beta distributions. (A: perturbation test for positive confounding; B: perturbation test for negative confounding; C: perturbation adjustment for positive confounding; D: perturbation adjustment for negative confounding; solid lines: a 50:50 mixture of$f_{\text{PV}}=0.06$ and $f_{\text{PV}}=0.04$; dotted lines: a 50:50 mixture of$f_{\text{PV}}=0.035$ and $f_{\text{PV}}=0.015$; horizontal lines: standardized relative risks).

Figure S2. Results of the perturbation analysis for the hypothetical population in Table 1 with dependent perturbation variables (A: perturbation test for positive confounding; B: perturbation test for negative confounding; C: perturbation adjustment for positive confounding; D: perturbation adjustment for negative confounding; solid lines: $f_{\text{PV}}=0.05$; dotted lines: $f_{\text{PV}}=0.025$; horizontal lines: standardized relative risks). The values of the perturbation variables within a subject are assumed to arise from a first-order Markov chain with an odds ratio between successive perturbation variables of 10.0.

Figure S3. Results of the perturbation analysis for the hypothetical population in Table 1 when 15% of the perturbation variables are pure noises (A: perturbation test for positive confounding; B: perturbation test for negative confounding; C: perturbation adjustment for positive confounding; D: perturbation adjustment for negative confounding; solid lines: $f_{\text{PV}}=0.05$; dotted lines: $f_{\text{PV}}=0.025$; horizontal lines: standardized relative risks).


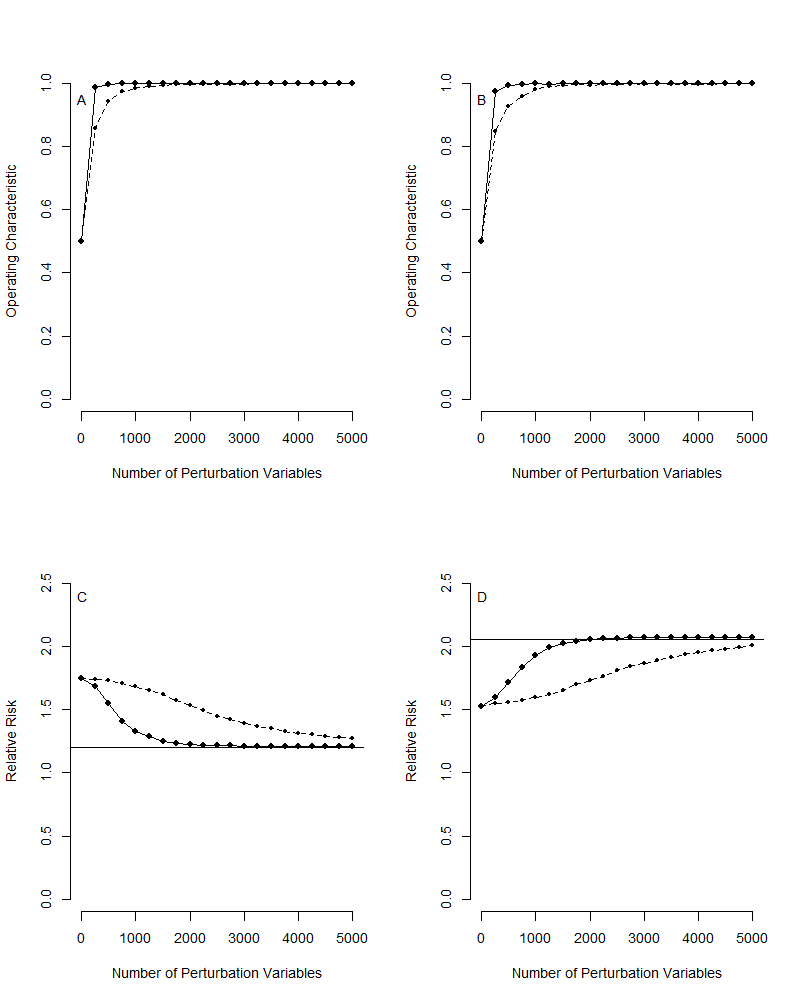
Figure S1.


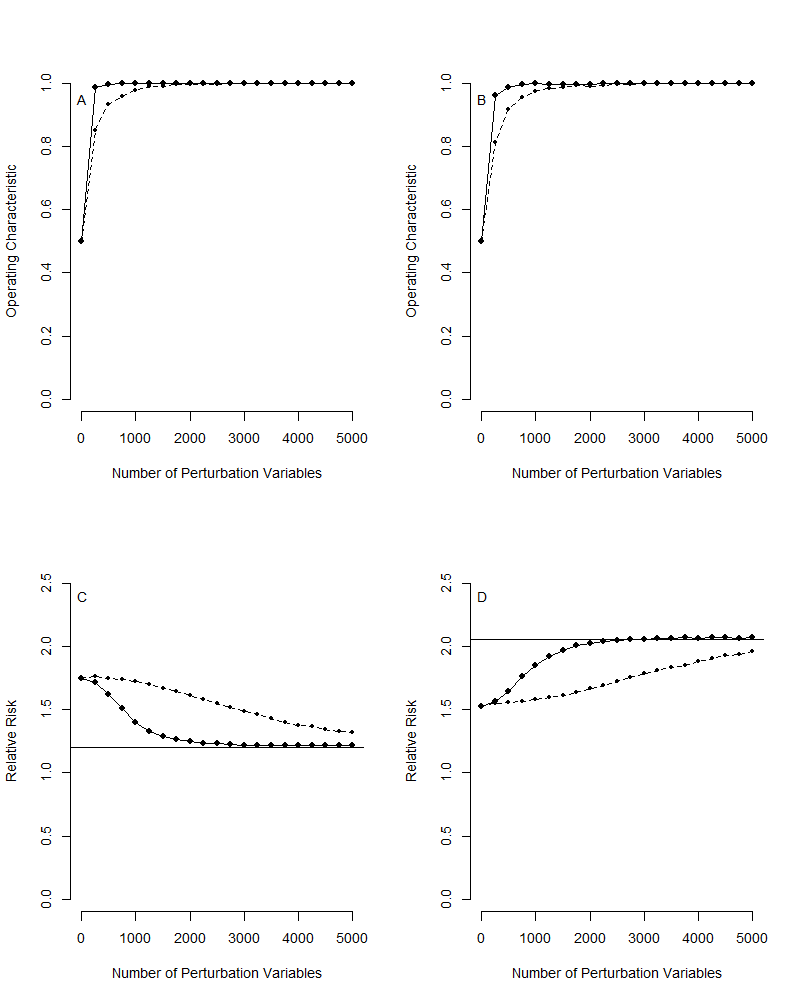


Figure S2.


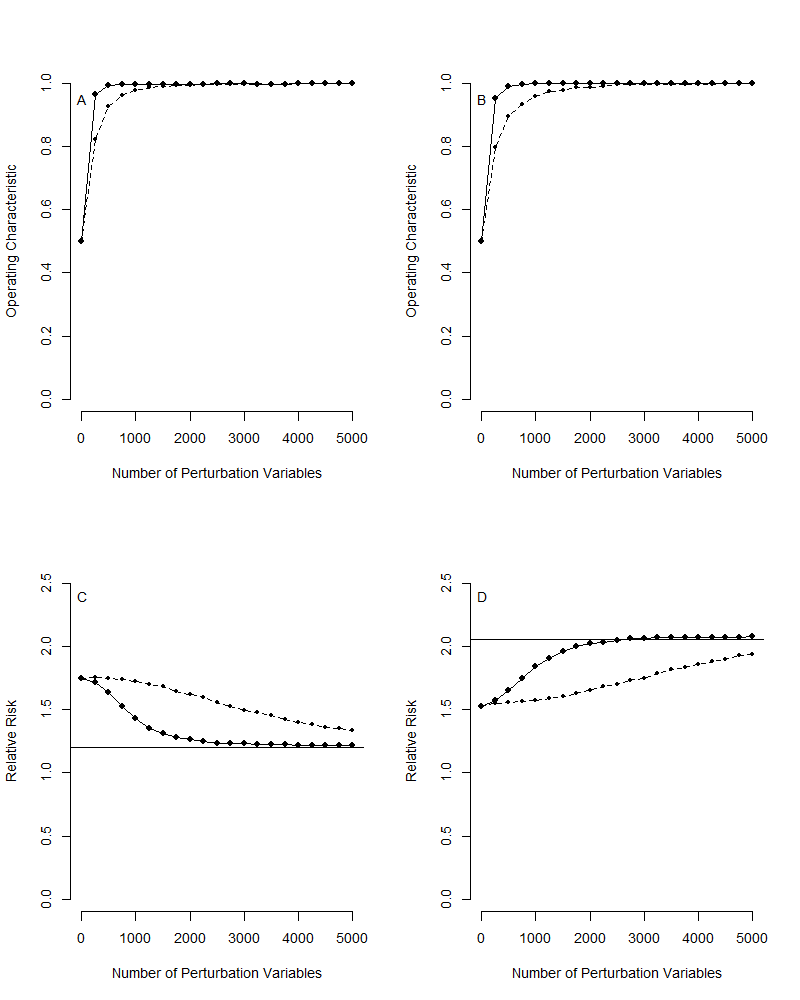


Figure S3.
